# Discovery, Biological Evaluation and Binding Mode Investigation of Novel Butyrylcholinesterase Inhibitors Through Hybrid Virtual Screening

**DOI:** 10.3390/molecules30102093

**Published:** 2025-05-08

**Authors:** Lizi Li, Puchen Zhao, Can Yang, Qin Yin, Na Wang, Yan Liu, Yanfang Li

**Affiliations:** School of Chemical Engineering, Sichuan University, Chengdu 610065, China

**Keywords:** butyrylcholinesterase inhibitors, virtual screening, machine learning, molecular docking, biological evaluation, binding mode analysis, molecule dynamics simulations

## Abstract

Butyrylcholinesterase (BChE), plays a critical role in alleviating the symptoms of Alzheimer’s disease (AD) by regulating acetylcholine levels, emerging as an attractive target for AD treatment. This study employed a quantitative structure–activity relationship (QSAR) model based on ECFP4 molecular fingerprints with several machine learning algorithms (XGBoost, RF, SVM, KNN), among which the XGBoost model showed the best performance (AUC = 0.9740). A hybrid strategy integrating ligand- and structure-based virtual screening identified 12 hits from the Topscience core database, three of which were identified for the first time. Among them, piboserod and Rotigotine demonstrated the best BChE inhibitory potency (IC_50_ = 15.33 μM and 12.76 μM, respectively) and exhibited favorable safety profiles as well as neuroprotective effects in vitro. Notably, Rotigotine, a marketed drug, was newly recognized for its anti-AD potential, with further enzyme kinetic analyses revealing that it acts as a mixed-type inhibitor in a non-competitive mode. Fluorescence spectroscopy, molecular docking, and molecular dynamics simulations further clarified their binding modes and stability. This study provides an innovative screening strategy for the discovery of BChE inhibitors, which not only identifies promising drug candidates for the treatment of AD but also demonstrates the potential of machine learning in drug discovery.

## 1. Introduction

Alzheimer’s Disease (AD) is a progressive neurodegenerative disorder marked by memory and cognitive decline, posing a growing public health challenge as its prevalence rises with an aging population [1,2]. Current therapeutic options for AD in clinical practice, such as cholinesterase inhibitors (ChEI) and N-Methyl-D-aspartic acid receptor antagonists, provide symptomatic relief but fail to halt or reverse disease progression and often cause side effects like gastrointestinal disturbances and cardiovascular issues [3]. Therefore, developing safer and more effective therapies is both clinically critical and socially valuable [4].

The pathogenesis of AD is complex, involving mechanisms like β-amyloid (Aβ) accumulation, tau protein hyperphosphorylation, neuroinflammation, oxidative stress, and cholinergic dysfunction [5,6]. Among these, Aβ aggregation and tau abnormalities are central hallmarks, while the cholinergic hypothesis is one of the earliest theories of AD pathogenesis [7,8]. The degeneration of cholinergic neurons not only directly impairs memory and learning but may also exacerbate other pathological processes, including tau phosphorylation, neuroinflammation, and neuronal apoptosis. Consequently, therapeutic strategies targeting the cholinergic system have emerged as a vital therapeutic target for AD [9].

It is widely known that acetylcholinesterase (AChE) and butyrylcholinesterase (BChE) are the primary enzymes degrading acetylcholine (ACh) [10]. Currently approved ChEIs, such as donepezil, galantamine, and rivastigmine, mainly target AChE. It is worth noting that they displayed limited efficacy, particularly in advanced AD, and notable side effects [11]. Recently, there has been growing interest in the potential of BChE inhibitors. While BChE plays a minor role in healthy individuals, its activity is significantly elevated in AD patients, especially during the advanced stages of the disease [12]. Research suggests that BChE not only contributes to acetylcholine degradation but may also be linked to key pathological features of AD, such as Aβ aggregation [13].

Conventional drug-discovery methods, such as high-throughput screening (HTS), require extensive compound libraries and significant experimental resources, making the process both time-consuming and expensive. In contrast, computer-aided drug design (CADD) utilizes computational simulations and algorithms to efficiently screen and optimize drug candidates, thereby reducing costs and accelerating the development process [14]. There are two approaches involved in CADD: ligand-based virtual screening (LBVS) and structure-based virtual screening (SBVS). The former builds quantitative structure-activity relationship (QSAR) models from known active compounds to predict the activity of new compounds, making it suitable when the target protein’s structure is unknown. Noticeably, its accuracy depends on the quality and diversity of known compounds and struggles to identify novel scaffolds [15]. The latter predicts ligand binding affinity using the three-dimensional structure of the target protein through molecular docking, providing insights into ligand-protein interactions and structure-activity relationships. Therefore, it requires high-quality target structures, accurate scoring functions, and significant computational resources. Hence, the combining strategy had been adopted and proven to improve the screening efficiency and accuracy [16,17]. Importantly, by analyzing the ADME/T properties (absorption, distribution, metabolism, excretion, and toxicity) of hits, CADD facilitates the selection of candidates with favorable pharmacokinetics and low toxicity, thereby enhancing the likelihood of successful drug development [18].

In the past decade, the rapid advancement of artificial intelligence (AI) technology has brought new momentum to CADD. It significantly shortens the drug-development timeline and reduces costs, demonstrating remarkable efficiency in processing large-scale datasets. Unlike conventional CADD methods, AI-based virtual screening offers notable advantages in predicting molecular properties due to its robust feature extraction and generalization capabilities [19,20].

For these attractive reasons, it has been applied to the field of BChE inhibitor discovery. Fang J et al. (2013) used support vector machine (SVM) and naive Bayes models to distinguish BChE inhibitors, experimentally validating their predictions and discovering new inhibitor scaffolds [21]. Malik AA et al. (2021) employed a random forest algorithm to develop a large-scale classification model, revealing key substructures affecting cholinesterase activity [22]. Ganeshpurkar A et al. (2021) applied a machine learning-based scoring function to identify martinyl cholinesterase inhibitors for drug screening [23]. Nguyen HD et al. (2023) utilized machine learning models, including Bayesian model averaging (BMA), artificial neural networks (ANN), multiple nonlinear regression (MNLR), and multiple linear regression (MLR), combined with molecular docking, to identify potential BChE inhibitors [24]. Meanwhile, Xu T et al. (2023) improved experimental hit rates and identified active AChE and BChE inhibitors through machine learning-based virtual screening [25]. These efforts underscore the promise of AI-driven approaches in accelerating BChE inhibitor research.

However, new problems arose, and limitations need to be addressed. First, the predictive performance of AI models heavily relies on the quality of training data [26]. Currently, the limited number of active compounds and imbalanced data distribution in public databases for BChE inhibitor screening hinder the models’ generalization ability and reduce prediction accuracy for novel compounds. Second, the lack of extensive in vitro and in vivo experimental validation, coupled with insufficient exploration of the action mechanisms of the identified inhibitors, restrict their practical applicability in further development process [27]. Additionally, limited consideration of drug formulation aspects, particularly the prediction of blood-brain barrier (BBB) permeability, hinders their effectiveness in targeting the central nervous system [28].

Therefore, this study applied a hybrid virtual screening strategy that integrated ligand- and structure-based methods, employed machine learning, and utilized various CADD techniques (such as molecular docking, ADME prediction, and BBB permeability assessment), to efficiently identify potential BChEIs with higher potency, which aimed to balance efficiency, accuracy, and drug availability. The activities and pharmacological properties of hits were further validated at both the enzyme and cellular levels. Additionally, fluorescence spectroscopy, molecular docking, and molecular dynamics simulations were used to clarify the binding mechanisms between the hits and BChE, providing guidance for future drug optimization. This research provides a useful virtual screening framework for BChE inhibitors (BChEIs) development and serves as a valuable reference for the discovery of therapeutic drugs for AD.

## 2. Results and Discussion

### 2.1. Machine Learning Modeling and Evaluation

#### 2.1.1. Model Training and Selection

Quantitative structure-activity relationship (QSAR) models offer a practical and efficient substitute for conventional wet lab experiments [29,30]. The machine learning method was adapted to build QSAR models for the prediction of compound activity and selectivity against AChE/BChE.

Data related to butyrylcholinesterase inhibitory activity from ChEMBL and associated activity reports were employed for model training. Following the implementation of preprocessing, a total of 711 small molecules were obtained, comprising 314 active compounds (IC_50_ < 100 nM) and 397 inactive compounds (IC_50_ > 10,000 nM). The molecules were randomly divided into a training set and a test set for model training and validation (7:3).

The ECFP4 fingerprint was generated by the RDKit package through smile conversion, a numerical representation of molecular structures that is suitable for machine learning. Regarding the high efficiency of ECFP4 in capturing local molecular features, this is conducive to the prediction of the interaction between biological targets, and its feature is a high-dimensional binary descriptor, which is suitable for training of binary classification machine learning models [31].

Four machine learning algorithms—Random Forest (RF), Support Vector Machine (SVM), K-Nearest Neighbors (KNN), and Extreme Gradient Boosting (XGBoost)—were employed to construct BChEIs activity prediction models, with hyperparameter optimization being carried out through gradient grid search. This optimization process included tuning parameters such as the number of estimators and maximum depth for RF, kernel type and regularization parameters for SVM, the number of neighbors and distance metrics for KNN, and learning rate and tree depth for XGBoost.

Final evaluation on a test set was conducted to choose the best-performing models. The Confusion Matrix-based and Receiver Operating Characteristic (ROC) Curve-based evaluation were used to compare the performance of different classification algorithms. Table 1 summarize the Accuracy and Area Under the Curve (AUC) score of each model under the optimal parameters. Although RF achieved slightly higher accuracy, the AUC score indicated that XGBoost demonstrated superior overall performance.

#### 2.1.2. Model Performance Evaluation

**Figure 1:**
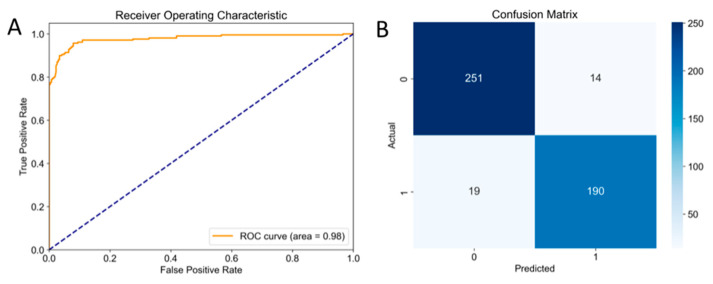
(**A**) The ROC Curve of XGBoost model. The dotted line in the figure represented the performance of the random classifier (AUC = 0.5); (**B**) The confusion matrix (math.) of XGBoost model, represented True Negative (TN) = 386, False Positive (FP) = 11, False Negative (FN) = 25, and True Positive (TP) = 289, which were the number of BChEIs predicted as BChEIs, non-BChEIs predicted as non-BChEIs, non-BChEIs predicted as BChEIs, BChEIs predicted as non-BChEIs, respectively.

To further evaluate the performance of the XGBoost model, the ROC curve and the confusion matrix were analyzed (Figure 1).

The ROC curve reflects the classification performance of the model under different thresholds [32]. It was observed from Figure 1A, it rose steeply and approached the upper left corner, indicating that the model was able to balance ‘high recall’ and ‘low false positives’ in most cases (i.e., the False Positive Rate was close to 0, and the True Positive Rate was close to 1). The AUC score of 0.9740 further substantiated the efficacy of the model, classifying it as performing at a high level of accuracy.

It should be noted that the AUC value is a useful summary statistic; it does not provide detailed insights into performance at specific thresholds. In this regard, multiple evaluation indicators including Precision, Recall, Specificity, F1 Score, and Matthews Correlation Coefficient (MCC) were calculated based on the confusion matrix [33]. The results revealed that the model achieves a Precision of 96.33% and a Recall of 92.04%, indicating its accuracy in reducing false positives and identifying positive BChEIs, while the F1 score of 94.13% further verified a strong balance between precision and recall. The Specificity of 97.13% demonstrated the model’s effectiveness in correctly identifying non-BChEIs, with a notably low false positive rate. Meanwhile, the Matthews Correlation Coefficient (MCC) of 0.8976, which is close to 1, indicated that the model was highly balanced and displayed excellent stability in the prediction of BChEIs. Overall, the model delivered a high level of performance in the classification task, achieving an overall accuracy of 94.94% while maintaining high Precision, Recall, and Specificity.

In addition, cross-validation was utilized to test the robustness of a model, achieving high prediction accuracies of 0.8488. This work indicated that the model was unlikely to be overfitted, since the prediction accuracies were similar [34].

In short, BChEIs prediction model using the XGBoost method possessed the ability to screen potential hits from a wide range of chemical spaces. By using machine learning models, it was possible to omit the recognition of drug molecular features based on empirical knowledge, which made it easier to discover new potential effective molecules to a certain extent [35].

### 2.2. Virtual Screening Framework

**Figure 2:**
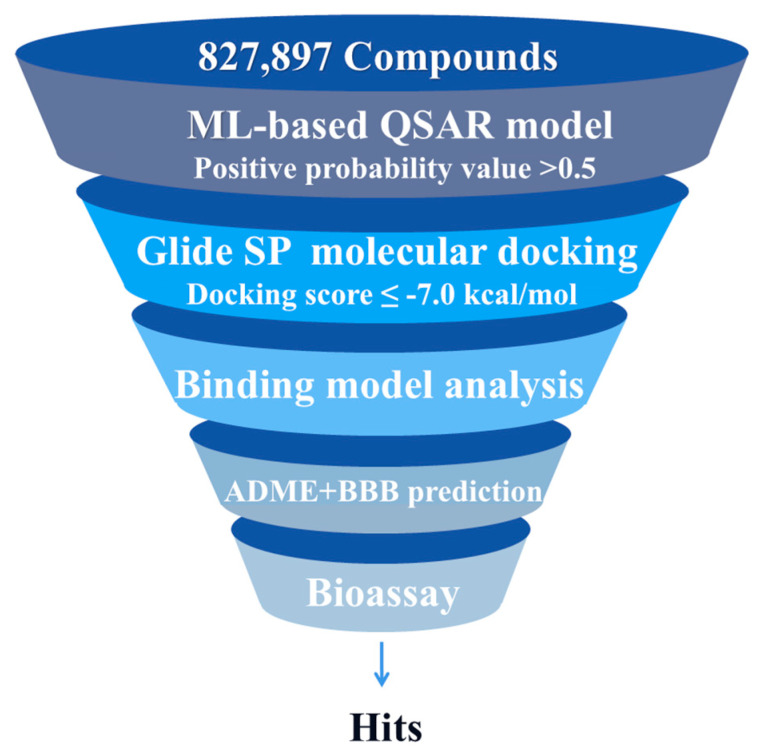
The virtual screening framework.

The integration of hybrid virtual screening technology in drug discovery has achieved significant advancement by combining the efficiency of ligand-based virtual screening (LBVS) with the precision of structure-based virtual screening (SBVS), thereby enhancing the accuracy and reliability of screening outcomes [16]. In this study, this strategy was employed to evaluate 827,897 molecules from a pre-cleaned sublibrary of the Topscience core database (https://www.tsbiochem.com/data-service/topscience-database, accessed on 7 May 2024). Figure 2 shows its framework, where multiple in silico techniques, (i.e., machine learning, molecular docking, ADME prediction, and BBB permeability) were successively applied to identify potential BChE inhibitors.

### 2.3. Hybrid Virtual Screening

**LBVS.** In Section 2.1, a statistically robust QSAR model was successfully developed for ligand-based virtual screening. The probability values (either positive or negative) were used to quantify the predicted activity, with a higher positive probability value indicating a greater chance to be a BChEls. The model was subsequently employed for high-throughput screening (HTS) of database, ranking from high to low according to their probability values. Only those with a value exceeding 0.5 were selected for further evaluation. A total of 129 molecules derived from this process were submitted for further molecular docking. Remarkably, the entire screening procedure was completed in approximately 20 min. This showed, the integration of the machine learning model reduced the molecular library to approximately one in a thousand candidates, screening efficiency improved significantly compared to traditional CADD techniques.

**SBVS.** To evaluate the accuracy of the molecular docking protocol, the selected protein (PDB ID: 5DYW) underwent a redocking study [36]. The co-crystal ligand *N*-((1-benzylpiperidin-3-yl) methyl)-N-(2-methoxyethyl) naphthalene-2-sulfonamide was redocked into the binding site of BChE (Figure S1A) to determine the RMSD of the initial and projected postures. The resulting RMSD value of 0.889 Å, which was less than 2 Å, confirmed the high precision of the docking method. Furthermore, Tacrine, Donepezil, and Galantamine were docked into the protein binding site (Figure S1B–D), yielding docking scores of −8.040, −7.914, and −7.142, respectively. The consistency of their docking scores reflected the comparable inhibitory potential of the three drugs and further validated the reliability of the docking approach.

The molecules identified from the preliminary screening were subjected to a preprocessing, and a total of 256 generated conformations were then docked into the active site of BChE, yielding a range of docking scores from −3.072 kcal·mol^−1^ to −11.13 kcal·mol^−1^. The hits were primarily selected according to their docking scores, with −7.000 kcal·mol^−1^ (referenced to the positive control) as the cutoff. Top-ranked molecules were further evaluated for the rationality of their conformations and their interactions with key amino acid residues. Consequently, 64 molecules were prioritized for further investigation based on docking scores and artificial assessment, while reported molecules were excluded from consideration.

**In silico prediction of drug-likeness.** The next prediction of the ADME properties and BBB permeability disclosed the drug-like features of hits. To eliminate potential failure hits, only molecules with CNS > 0, QPlogBB > −3, and BBB Score > 3 were selected, which indicated adequate CNS activity and BBB permeability [37]. Gastrointestinal absorption was further optimized by evaluating the intestinal blood barrier (QPPCaco) and the human oral absorption (HOA %), which were both identified as key indicators. Cut-off values of 500 for QPPCaco and 70 for HOA % were set based on recommended values and the positive control (Table S1). Furthermore, Lipinski’s Rule of Five and Jorgensen’s rule of three were incorporated into the screening criteria. Following this, 12 potential hits with favorable druggability were chosen for further biological evaluation.

### 2.4. Hits Identification

Table 2 list a summary of the structures of 12 hits, along with their relevant values and data obtained during the screening process. Predicted ADME values and BBB scores are detailed in Table S1, which included only the compounds confirmed to exhibit inhibitory activity, serving as a basis for further activity tests. Notably, two marketed drugs (Indacaterol and Rotigotine) were identified, highlighting the directionality and practicality of the screening methodology. Unlike common screening strategies, the current strategy not only evaluated the binding affinity between the target and the ligand but also emphasized the safety and clinical translational potential of candidate molecules by incorporating comprehensive druggability assessments.

### 2.5. In Vitro Biological Activity Evaluation

#### 2.5.1. Enzyme Inhibition

Twelve hits were purchased commercially and evaluated for inhibitory potency against BChE in vitro; Tacrine, Donepezil and Galantamine served as positive controls. Four hits (Table 3) exhibited inhibitory activity with over 50% inhibition at a concentration of 50 µM. Among them, piboserod, Metergoline, and Rotigotine were identified as BChE inhibitors for the first time, with IC_50_ values of 15.33 μM, 18.36 μM, and 12.76 μM, respectively (see Figure S2 for detailed inhibition curves). It was noteworthy that Rotigotine, a marketed neurological disorder drug primarily used in Parkinson’s disease and idiopathic restless legs syndrome, was first identified as a BChE inhibitor. Several studies concerning its potential application in AD treatment were conducted, which revealed its potential effects on central cholinergic transmission in AD patients [38]. In addition, it could reduce neuroinflammation, oxidative stress, and acetylcholinesterase activity in mouse models, resulting in improved cognitive deficits [39,40]. Subsequently, piboserod, Metergoline, and Rotigotine were selected for cell studies and enzyme kinetics assays, and the best-performing compounds were submitted for binding mode investigation.

#### 2.5.2. Toxicity and Neuroprotective Effect Studies

**Figure 3:**
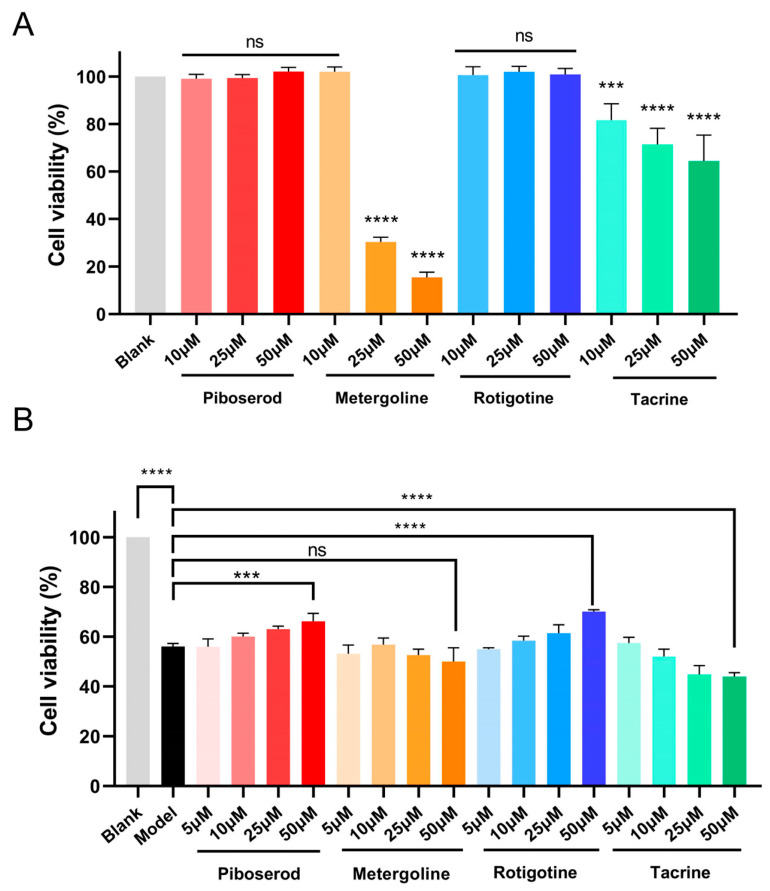
(**A**) Toxicity of piboserod, Metergoline, and Rotigotine on PC-12 cells at 10, 25, and 50 μM; (**B**) Protective effect of piboserod, Metergoline, and Rotigotine against hydrogen peroxide-induced PC-12 cell damage. (The data are expressed as mean ± SD of three independent experiments. *** means *p* < 0.001; **** means *p* < 0.0001; ns means no significant difference).

In view of favorable ADME predictive properties of three hits, their neurotoxicity was evaluated in PC-12 cells. Cell viability was measured using the MTT assay, with Tacrine serving as a reference control. As shown in Figure 3A, Tacrine exhibited a dose-dependent increase in toxicity, resulting in only 65% relative cell viability at 50 μM. In contrast, piboserod and Rotigotine demonstrated no neurotoxicity across all tested concentrations (10 μM, 20 μM, and 50 μM), maintaining cell viability above 95%. Metergoline displayed no neurotoxicity at the lowest concentration (10 μM) but showed higher cytotoxicity than that of Tacrine at maximum concentration. These results suggested that piboserod and Rotigotine exhibit superior low-toxicity properties in vitro compared to the positive drug tacrine, while Metergoline demonstrated an excellent safety profile at low doses.

In addition to the ACh, oxidative stress disorders are frequently observed in the brains of AD patients. The overproduction of reactive oxygen species (ROS) can perpetuate brain damage and further exacerbate disease progression [41]. In order to evaluate the neuroprotective effects of three hits against oxidative damage, a hydrogen peroxide-induced injury model in PC-12 cells was established. Cell viability was assessed using the MTT assay, with Tacrine as the reference control. As shown in Figure 3B, compared with the blank group, the cell viability of PC-12 cells treated with hydrogen peroxide (the model group) was reduced to 56%. Encouragingly, both piboserod and Rotigotine demonstrated significant neuroprotective activities within the concentration range of 5 to 50 μM (cell viability of 66% and 70% at 50 μM, respectively), and the protective effects were dose-dependent. This indicated that both of them possessed the potential to protect PC-12 against hydrogen peroxide damage; in particular, Rotigotine demonstrated a greater protective effect. Noticeably, Metergoline also showed modest neuroprotective effects at low concentrations (5–10 μM), but its protective capacity diminished at higher concentrations due to the emergence of cytotoxicity. Therefore, piboserod and Rotigotine were chosen for further investigation.

#### 2.5.3. Enzyme Kinetics Analysis

**Figure 4:**
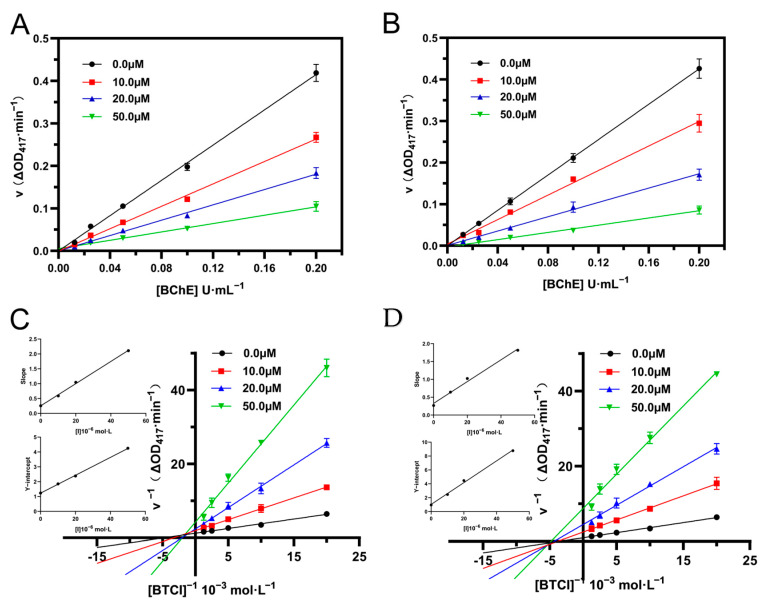
(**A**) Plots of ν vs. [BChE] for piboserod; (**B**) Plots of ν vs. [BChE] for Rotigotine; Lineweaver-Burk plots of inhibition of piboserod (**C**) and Rotigotine (**D**), the secondary plots represent the Slope vs. [inhibitors] plot and the Y-intercept vs. [inhibitors] plot from top to bottom, respectively.

Kinetic studies were performed to clarify the inhibition mechanism of piboserod and Rotigotine [42,43]. Figure 4A,B illustrates the relationship between BChE concentration ([BChE]) and catalytic rate (v) at different inhibitor concentrations ([I]). All fitted curves passed through the origin, with slopes decreasing as inhibitor concentration increased, which confirmed that two hits reversibly inhibit BChE. Lineweaver–Burk plots were used to characterize their inhibition types [44]. As shown in Figure 4C, the curves of piboserod intersected in the second quadrant, with both the horizontal intercept (−1/K_m_) and vertical intercept (1/V_max_) increasing with higher inhibitor concentrations, indicating mixed-type inhibition of BChE. In addition, the curves of Rotigotine (Figure 4D) showed a decrease in V_max_ without changes in K_m_ as inhibitor concentration increased, consistent with non-competitive inhibition. The inhibition constant (K_i_) values were calculated based on Lineweaver–Burk secondary plots (Figure 4C,D). The Ki values were 10.73 μM for piboserod and 6.76 μM for Rotigotine, which were compatible with the results of enzyme inhibition experiments. Linear fitting of the secondary plots suggested that two hits likely interacted with BChE at one or a specific class of binding sites. Moreover, α value of piboserod (the ratio of non-competitive to competitive inhibition constants), was calculated to be 3.01, indicating stronger affinity to the free enzyme (α > 1) than to the enzyme-substrate complex [45].

### 2.6. Binding Mode Investigation

#### 2.6.1. Fluorescence Quenching Experiments

**Figure 5:**
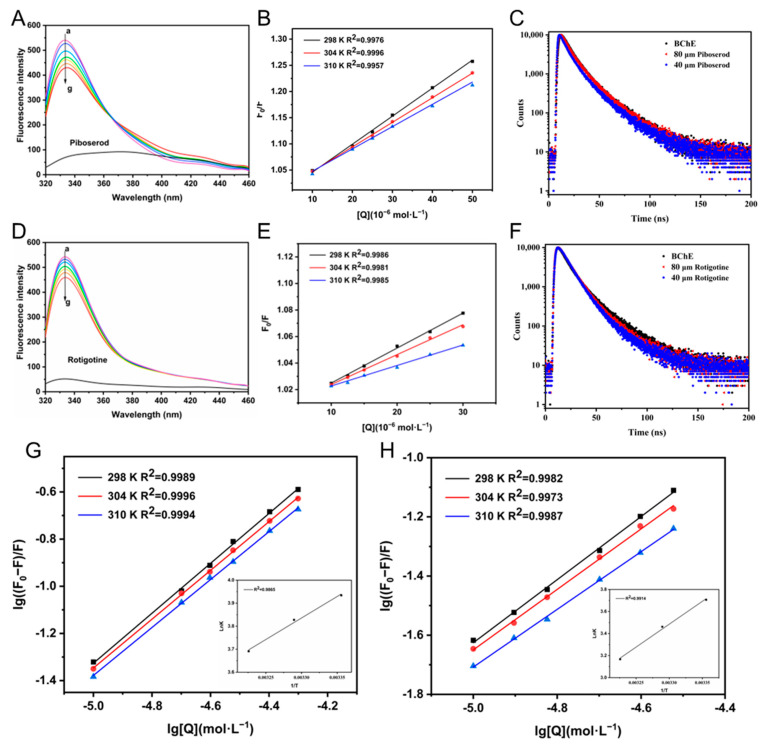
(**A**) Fluorescence-quenching spectra of BChE (5 U·mL^−1^) in the presence of piboserod, with varying concentrations at 298 K, c(piboserod) = 0, 10, 20, 25, 30, 40, and 50 μM for curves a → g, respectively; (**B**) The Stern-Volmer plots for the fluorescence quenching of BChE by piboserod at 298, 304, and 310 K; (**C**) Time-resolved fluorescence decay curve of the piboserod-BChE system; (**D**) Fluorescence quenching spectra of BChE (5 U·mL^−1^) in the presence of Rotigotine, with varying concentrations at 298 K, c(Rotigotine) = 0, 10, 12, 15, 20, 25, and 30 μM for curves a → g, respectively; (**E**) The Stern-Volmer plots for the fluorescence quenching of BChE by Rotigotine at 298, 304, and 310 K; (**F**) Time-resolved fluorescence decay curve of the Rotigotine-BChE system; The Stern-Volmer bilogarithmic plots for the fluorescence quenching of BChE by piboserod (**G**) and Rotigotine (**H**) at 298, 304, and 310 K, the secondary plots represent Van’t Hoff plots for the interaction of each with BChE.

Fluorescence-quenching experiments at 280 nm excitation were performed to unveil the binding mechanisms of piboserod and Rotigotine. Emission from BChE arose from tryptophan (Trp), tyrosine (Tyr), and phenylalanine (Phe) residues, and a distinct peak at 340 ± 10 nm primarily attributed to Trp [46]. As shown in Figure 5A,D, both exhibited weak intrinsic fluorescence at high concentrations (50 μM), which did not affect the main emission peak. The fluorescence intensity of BChE at 335 nm gradually diminished with rising concentrations (curves a–h), while the maximum emission appeared no obvious shift. These results suggest both compounds interacted with BChE effectively.

Then, the Stern–Volmer Equation (1) was applied to analyze their quenching mechanisms. As shown in Figure 5B,E, the linear relationship between F_0_/F and [Q] indicates a single quenching process, while he Stern–Volmer constants (K_sv_) for both inhibitors decrease with rising temperature (Table 4). At 298 K, the quenching rate constants (K_q_) for each were 5.25 × 10^11^ and 2.64 × 10^11^ L·mol^−1^·s^−1^, significantly exceeding the maximum diffusion collision quenching constant (2.0 × 10^10^ L·mol^−1^·s^−1^), which suggested a static quenching mechanism driven by complex formation [45]. Time-resolved fluorescence data (Figure 5C,F) further support these observations, since fluorescence decay curves were consistent across varying concentrations of the compounds, indicating that BChE fluorescence lifetimes remained unaffected and confirming the static quenching mechanism.

Their binding constants (K_a_) and binding sites (n) (Table 4) with BChE were calculated using the double logarithmic Equation (2) (Figure 5G,H). The K_a_ values of both inhibitors dropped progressively as the temperature grew from 298 to 310 K, suggesting reduced stability of the complexes at elevated temperatures. This observation aligns with an exothermic, enthalpy-driven binding process [47]. Meanwhile, the K_a_ values, on the order of 10^4^ L·mol^−1^, indicate strong binding affinity of piboserod to BChE, in contrast to the relatively lower binding affinity of Rotigotine. In line with enzyme inhibition kinetics, piboserod acts as a mixed competitive inhibitor, partially influenced by the substrate, while Rotigotine, a non-competitive inhibitor, efficiently inhibits without competing with the substrate. Thus, Rotigotine, despite its weaker binding, exhibited a higher IC_50_ value. Additionally, the binding site number (n) remained close to 1 across all temperatures, indicating that both inhibitors primarily interact with a single binding site on BChE [48]. This finding is in strong agreement with information obtained using the Lineweaver–Burk plot analysis:

(1) $$\frac{F_{0}}{F}=1\;+\;K_{\mathrm{sv}}[Q]1\;+\;K_{q}\tau_{0}\left[Q\right]$$

(2) $$\log\frac{F_{0}-F}{F}=\log K_{a}\;+\;n\log\left[Q\right]$$

where F_0_ and F are the fluorescence intensity in the absence and presence of quencher, respectively; K_sv_ and K_q_ represent the Stern–Volmer quenching constant and fluorescence quenching rate constant; τ_0_ (10^−8^ s) denotes the average fluorescence lifetime of free BChE; [Q] is the concentration of quencher; K_a_ represents the binding constant.

Thermodynamic parameter analysis offers valuable insights into the predominant interaction forces governing the binding process between ligand and protein, which may encompass hydrogen bonding, electrostatic interactions, hydrophobic effects, van der Waals forces, and π-effects [49]. By applying the Van’t Hoff Equation (3) and the Gibbs–Helmholtz Equation (4), the thermodynamic parameters—including change (ΔS), enthalpy change (ΔH), and Gibbs free energy change (ΔG)—for the interactions of piboserod and Rotigotine at various temperatures. The Van’t Hoff plots are illustrated in Figure 5G and 5H (the secondary plots), with the computed results summarized in Table 4. The significantly negative ΔH value (|ΔH| > |TΔS|) and the negative ΔS value suggested that the ligand–BChE binding is predominantly driven by enthalpic contributions, likely mediated by hydrogen bonding or electrostatic interactions. Furthermore, the negative ΔG value confirms the thermodynamic spontaneity of the binding process [47].

(3) $$\ln K_{a}=-\frac{\Delta H}{\mathrm{RT}}+\frac{\Delta S}{R}\;$$

(4) ΔG=ΔH-TΔS

where R is the gas constant (8.314 J/mol^−1^·K^−1^), ∆H and ∆S are regarded as a constant.

#### 2.6.2. Three-Dimensional Fluorescence Spectroscopy

**Figure 6:**
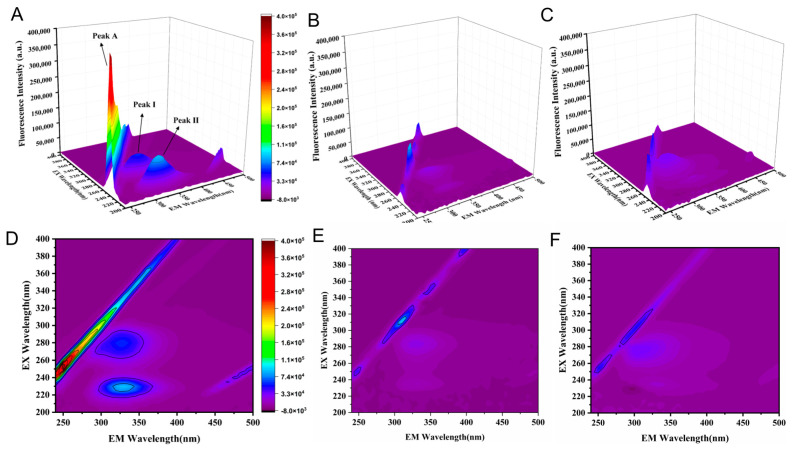
3D fluorescence spectra of BchE (**A**), BChE–piboserod system (**B**) and BChE–Rotigotine system (**C**), c(BChE) = 0.2 U·mL^−1^, c(piboserod) = 50 μM, c(Rotigotine) = 50 μM; Contour plots of 3D fluorescence spectra of BchE (**D**), BChE–piboserod system (**E**) and BChE–Rotigotine system (**F**).

The effects of two inhibitors on BChE conformation and the microenvironment of amino acid residues were assessed using three-dimensional fluorescence spectra for free BChE and its ligand complexes. The fluorescence spectral changes upon ligand binding are illustrated in Figure 6. BChE exhibits three primary peaks: peak A, the Rayleigh scattering peak (λex = λem) (Figure 6A), which is associated with the uniformity and diameter of BChE; peak I, corresponding to the spectral characteristics of tryptophan and tyrosine residues; and peak II, reflecting changes in the protein’s microenvironment [50]. Upon ligand binding (Figure 6B,C), peak A fluorescence intensity decreased significantly, suggesting a more compact protein conformation induced by complex formation, altering BChE′s optical properties. Similarly, fluorescence intensities of peaks I and II were reduced, with piboserod causing a more pronounced quenching effect. These phenomena indicated that both ligands bind to BChE, altering the fluorescence properties and microenvironment of tryptophan and tyrosine residues. Notably, piboserod exhibited a more substantial impact, indicating a stronger disturbance of fluorophore behavior upon binding.

#### 2.6.3. Molecular Docking

Molecular docking analysis was performed to elucidate the binding sites and interaction forces of piboserod and Rotigotine with BChE. It has been revealed that the active cavity of BChE consists of four key regions: the peripheral anion site (mainly Asp70), the choline binding pocket (mainly Trp82), the acyl binding pocket (mainly Trp231), and the catalytic active site (catalytic triad of Ser198, His438, and Glu325). Among these, Trp82 and Trp231 are identified as pivotal residues for ligand binding [51].

**Figure 7:**
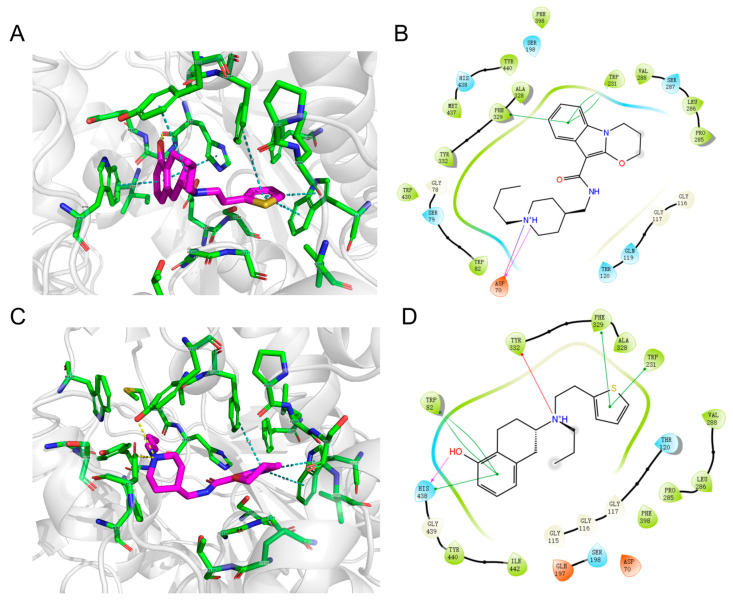
Docking interaction plots of piboserod (**A**) and Rotigotine (**C**) with active site residues of BChE (PDB ID 5DYW), Interactions included non-covalent bonds, π interaction, the hydrogen bonds in yellow, π-π stacking in blue, π-cation in green; 2D interaction schematic of piboserod (**B**) and Rotigotine (**D**) with BChE. Hydrogen bonds were shown as purple lines, salt bridge was shown as a pink violet gradient, π-π stacking interactions were in green lines, and π-ion bonds were in red lines.

Figure 7A,B showed that the piperidine ring of piboserod forms a salt bridge with the peripheral anionic site Asp70, further stabilized by hydrogen bonding. This strong interaction likely drives piboserod binding, consistent with previous findings from fluorescence quenching experiments. Additionally, the indole’s benzene ring formed three π-π stacking interactions with Trp231 and Phe329 in the acyl binding pocket, potentially contributing to the stabilization of piboserod at the active site of BChE.

Figure 7C,D illustrated the binding of Rotigotine to BChE, with its tetrahydronaphthalene moiety deep into the cavity, forming multiple interactions with the choline binding pocket and catalytic active site, involving a hydrogen bond between the phenol group and His438, and three π-π stacking interactions between the benzene ring and His438 and Trp82. Among them, hydrogen bond is the main binding force of Rotigotine, which is also in accordance with the inferences obtained in the above investigations. Similar to piboserod, the thiophene ring of Rotigotine also forms two π-π stacking interactions with residues Trp231 and Phe329 at the acyl binding pocket, which may be correlated with the potent inhibitory effect of both. Most importantly, a π-ion interaction between the amine group and Tyr332 further stabilizes the complex.

This fact pointed out that piboserod and Rotigotine share a certain degree of similarities in their binding mechanisms, and Rotigotine’s superior BChE inhibitory activity is potentially attributed to its interactions with His438 and Trp82. Unlike piboserod, which involves one tryptophan (Trp) residue, Rotigotine engages two Trp residues and one tyrosine (Tyr), suggesting a stronger influence on the fluorophore. This aligns with findings from 3D fluorescence spectroscopy studies.

#### 2.6.4. Molecular Dynamics Simulation

Molecular docking can only provide static binding modes. It should be stressed that binding between ligand and receptor is essentially a dynamic phenomenon. in view of this, the binding stability of piboserod–BChE and Rotigotine–BChE complexes and their interaction mechanisms were further analyzed using molecular dynamics simulation techniques.

**Figure 8:**
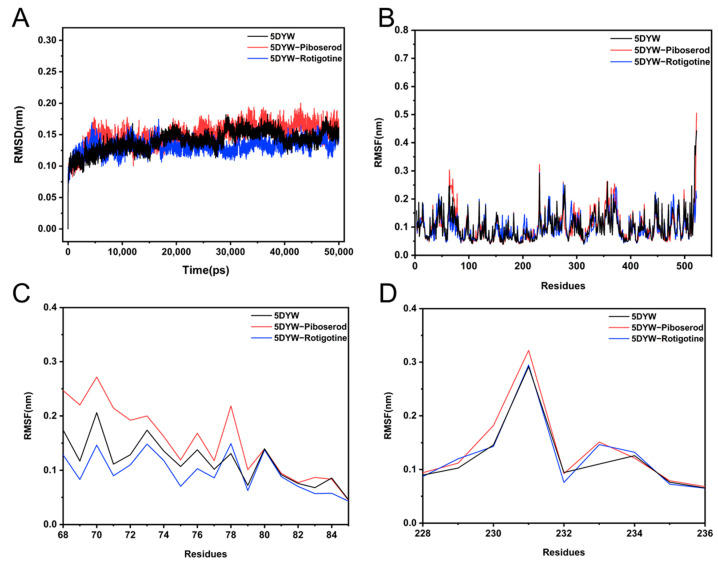
(**A**) RMSD analysis for piboserod-BChE and Rotigotine-BChE complexes; (**B**) RMSF plot of each residue in piboserod-BChE and Rotigotine-BChE complexes; (**C**,**D**) RMSF plot of the key residues in complexes piboserod-BChE and Rotigotine-BChE obtained from the full RMSF plot.

The stability of the system was assessed using the root mean square deviation (RMSD) of the protein backbone atoms, where lower RMSD values indicate greater stability of the complex [52]. As shown in Figure 8A, during the 50 ns simulation, the RMSD values for the free BChE and the two BChE–inhibitor systems remained below 0.20 nm and plateaued after approximately 10 ns, suggesting that the systems had achieved stable conformations. Following stabilization, the RMSD values fluctuated within the range of 0.10 to 0.20 nm. The BChE–piboserod system exhibited an RMSD value that converged to approximately 0.18 nm, slightly higher than the 0.15 nm observed for the free BChE, indicating that piboserod binding induced minor conformational disturbances. In contrast, the RMSD value for the BChE–Rotigotine system converged to approximately 0.12 nm, suggesting that its binding may result in a more stable overall protein conformation.

The root mean square fluctuation (RMSF) was employed to evaluate the amplitude of residue fluctuations and regional flexibility, with higher RMSF values indicating greater local fluctuations [52]. As shown in Figure 8B, the RMSF curves of the three systems exhibited a consistent overall trend, and no significant conformational changes were observed. With the exception of the C-terminus of the BChE protein, the RMSF values for most residues remained low (<0.2 nm), indicating that the systems were stable and exhibited considerable rigidity. However, slight fluctuations in RMSF values were observed in certain regions (Figure 8C), primarily localized to the active regions of the protein, which aligns with findings reported in previous literature. Compared to the free BChE, piboserod (red curve) induced a modest increase in RMSF at residues 68–85 and caused more pronounced fluctuations in the highly flexible region near residue 231, likely due to its interaction with Asp70 and Trp231, which may alter the local flexibility. Conversely, Rotigotine (blue curve) led to a reduction in RMSF values near residues Trp82 and Trp231, suggesting that its binding may enhance the protein’s stability and rigidity, consistent with those obtained with molecular docking.

**Figure 9:**
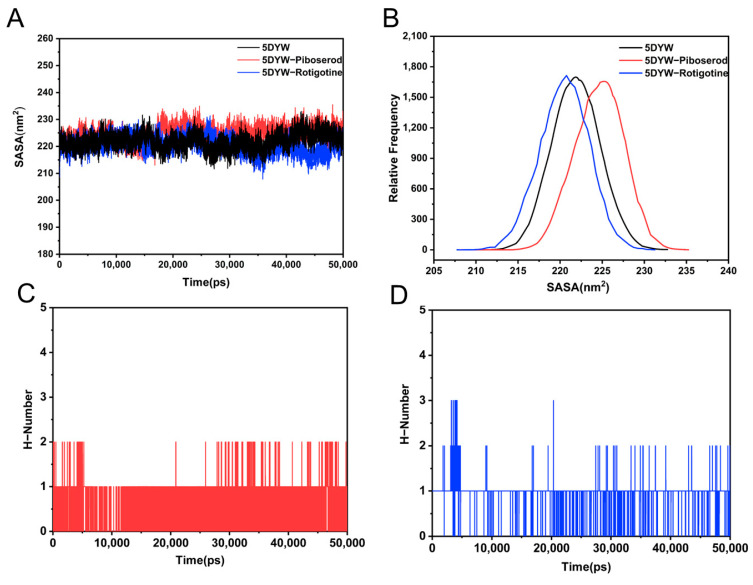
Time course (**A**) and relative distribution frequency (**B**) of SASA in piboserod-BChE and Rotigotine-BChE complexes; Number of hydrogen bonds between BChE and piboserod (**C**) and Rotigotine (**D**).

To further assess changes in the overall conformation of the system, solvent accessible surface area (SASA) analysis was conducted [52]. As shown in Figure 9A, the SASA values of the three systems exhibited small fluctuations within a similar range throughout the 50 ns simulation, remaining between 210 and 230 nm^2^. This indicates that the overall conformation of the systems remained stable without significant alterations. The statistical analysis of the relative frequency distribution (Figure 9B) revealed that the mean SASA value of the piboserod–BChE complex was notably higher than that of the free BChE. This increase is likely attributed to local relaxation of the protein induced by piboserod binding, resulting in an expanded solvent accessible surface area. In contrast, the average SASA value of the Rotigotine–BChE complex was slightly lower, suggesting that its binding may render the protein locally more compact.

Previous studies have suggested that the binding of piboserod and Rotigotine to BChE is primarily mediated by hydrogen bonding and salt-bridge interactions. To further investigate binding stability, the changes in the number of hydrogen bonds between the inhibitors and the protein were quantified during the molecular dynamic simulations. The results indicated that the number of hydrogen bonds between piboserod and BChE fluctuated between 0 and 1 (Figure 9C), reflecting dynamic behavior characterized by repeated bond formation and dissociation. This may be attributed to the high flexibility of the Asp70 residue or alterations in geometric conditions, which could weaken the synergistic effects of the salt bridge and hydrogen bonds. In contrast, the number of hydrogen bonds between Rotigotine and BChE remained consistently around 1 (Figure 9D), suggesting a more stable binding interaction. This observation further supports the conclusion that Rotigotine forms a stable association with BChE.

## 3. Materials and Methods

### 3.1. Data Source

The training dataset was derived from molecular information and activity data of the human-derived BChE enzyme (ChEMBL ID: CHEMBL1914) in the ChEMBL database (https://www.ebi.ac.uk/chembl/, accessed on 4 July 2023) [53]. First, duplicate removal and null value elimination operations were carried out. The activity data were evaluated based on the IC_50_ values. To enable the model to better distinguish the activity of compounds, those with IC_50_ values less than 1000 nanomoles were regarded as active compounds and labeled as 1, while those with IC_50_ values greater than 100,000 nanomoles were considered inactive compounds and labeled as 0. The data of the low-activity compounds in-between were deleted. Using the RDKit toolkit, molecular formats like mol were converted to SMILES, then transformed into ECFP4 fingerprints using RDKit’s ‘GetMorganFingerprintAsBitVect’ function (mol, radius = 2, nBits = 2048) [54]. The 2048-bit molecular fingerprints served as features to create the training dataset, which was split into training and testing sets.

The drug-like subset of the existing Topscience core database was selected for screening (https://www.tsbiochem.com/data-service/topscience-database, accessed on 7 May 2024). The database was pre-cleaned according to Lipinski’s Rule of Five by defining functions and using the NumPy package [55]. Following the same data processing methods as the training dataset, a screening library with identical features was obtained.

### 3.2. Machine Learning Modeling

RF, KNN, and SVM models were trained using the scikit-learn package, and an XGBoost model was trained using the XGBoost package [56,57]. Next, the training set was used to train the model. Model performance strongly depends on the selection of hyperparameters, so GridSearchCV was used for hyperparameter search [58]. Then, the test set and validation were used to evaluate the model performance, and the classification problem could use metrics such as accuracy, precision, recall, and so on. Scikit-learn provides a rich set of evaluation functions. At last, the established model was applied for high-throughput screening of the library, and probability predictions were generated.

### 3.3. Machine Learning Valuation

The ROC curve is a graphical representation that illustrates the performance of a classification model across various classification thresholds. It visually demonstrates the trade-off between the true positive rate (sensitivity) and the false positive rate (1-specificity) at different threshold settings. The Area Under the Curve (AUC) score is commonly used as a performance metric, particularly for imbalanced datasets, as it provides an intuitive and comprehensive measure of a model’s classification capability. The AUC score is particularly valuable for comparing the performance of multiple models. A higher AUC, closer to 1, indicates superior model performance, while an AUC of 0.5 suggests that the model performs no better than random guessing [32].

The confusion matrix is a fundamental evaluation tool for classification problems, presenting the distribution of a model’s prediction results in a matrix format. It contains four key metrics: True Positive (TP), True Negative (TN), False Positive (FP), and False Negative (FN). Various evaluation metrics can be derived from the confusion matrix to assess model performance.

Precision measures the accuracy of the model in predicting positive classes, indicating the proportion of correctly identified positive predictions out of all predicted positives. Recall, also known as sensitivity, evaluates the model’s ability to identify positive samples, with higher recall corresponding to a lower missed diagnosis rate. Specificity assesses the model’s ability to correctly identify negative samples, where higher specificity corresponds to a lower misdiagnosis rate. The F1 Score, being the harmonic mean of Precision and Recall, provides a balanced measure that considers both metrics simultaneously. The Matthews Correlation Coefficient (MCC) is a robust, comprehensive metric for evaluating classification models, accounting for the full relationship between TP, TN, FP, and FN. Its value ranges from −1 to 1, where 1 indicates perfect classification, 0 represents random classification, and −1 denotes complete misclassification. The MCC is particularly useful for imbalanced datasets, as it provides a more holistic evaluation of model performance [59]. Each parameter was calculated by the following Equations (5)–(9).

(5) $$\mathrm{Precision}=\frac{\mathrm{TP}}{\mathrm{TP}+\mathrm{FP}}$$

(6) $$\mathrm{Recall}=\frac{\mathrm{TP}}{\mathrm{TP}+\mathrm{FN}}$$

(7) $$\mathrm{Specificity}=\frac{\mathrm{TN}}{\mathrm{TN}+\mathrm{FP}}$$

(8) $$F1\;\mathrm{Score}=2\;\frac{\mathrm{Precision}\cdot \mathrm{Recall}}{\mathrm{Precision}+\mathrm{Recall}}$$

(9) $$\mathrm{MCC}=\frac{\mathrm{TN}\cdot \mathrm{TP}-\mathrm{FP}\cdot \mathrm{FN}}{\sqrt{\left(\mathrm{TN}+\mathrm{FP})+(\mathrm{TP}+\mathrm{FP})+(\mathrm{TP}+\mathrm{FN})+(\mathrm{TN}+\mathrm{FN}\right)}}$$

### 3.4. Molecular Docking

Following high-throughput screening using machine learning, a molecular docking study was performed using the Schrödinger Glide SP module (Schrödinger LLC, New York, NY, USA). First, the identified molecular structures were imported into Maestro to generate three-dimensional (3D) configurations and examine various conformations using the LigPrep module. Molecules in all conformations were treated at physiological pH (7.0 ± 2.0) and then minimised under the OPLS4 force field. Meanwhile, the crystal structure of BChE (PDB ID: 5DYW) in complex with active site inhibitors measured by the X-ray method was obtained from the RCSB Protein Data Bank (http://www.rcsb.org, accessed on 10 June 2024) and optimized using the Schrödinger Protein Preparation Wizard [60]. During that, Crystallographic water molecules were removed, missing hydrogen atoms were added, and zero-order metal and disulfide bonds were introduced. Then, the receptor grid was generated in the Receptor Grid Generation module (Schrödinger). The active site of protein was defined as a grid box with dimensions of 10 × 10 × 10 Å, the scaling factor was set to 0.8, and the partial charge cutoff was set to 0.15. The other parameters were set at the default values in Schrödinger. PyMOL (PyMOL 2016, New York, NY, USA) was used to offer the graphic display.

### 3.5. In Silico ADME Prediction and BBB Permeability

The ADME (absorption, distribution, metabolism, and excretion) characteristics are pivotal in the drug-discovery process [61]. Therefore, important physical descriptors and pharmacogically relevant properties of molecules were predicted using the QikProp module (Schrodinger), including the central nervous system activity (CNS), brain/blood partition coefficient (QPlogBB), percentage of human oral absorption (HOA%), intestinal blood barrier permeability (QPPCaco), and hydrogen bond donor/acceptor.

Moreover, blood-brain barrier (BBB) permeability is a critical factor for central nervous system (CNS) drugs. Thus, the blood-brain barrier score (BBB score) is used to estimate whether molecules can cross the central nervous system (CNS). The scoring mechanism is an algorithm-generated score with a predictive value of 0.86, which has been proved to be a reliable prediction method. Compounds with BBB scores ranging from 4 to 6 are typically classified as CNS drugs, while those with scores between 3 and 4 are considered intermediate molecules with ambiguous classification. Compounds with BBB scores below 3 are generally identified as non-CNS drugs [37].

### 3.6. BChE Inhibition Assay and Kinetic Study

The inhibitory activity of BChE was evaluated using 96-well plates on a microplate reader (Varioskan LUX, Thermo Fisher, Waltham, MA, USA) based on a modified Ellman method. Briefly, 25 μL of BChE solution (0.2 U·mL^−1^, Sigma, St. Louis, MO, USA, horse-serum, E.C. 3.1.1.8) was combined with 65 μL of phosphate buffer (PBS, 0.1 M, pH 8.0), 125 μL of 5,5′-dithio-bis(2-nitrobenzoic acid) (DTNB, 3 mM), and 10 μL of a test compound at varying concentrations. Small molecules (50 mM, Topscience) were initially dissolved in dimethyl sulfoxide (DMSO) and subsequently diluted to desired concentrations using PBS. The reaction mixture was preincubated at 37 °C for 20 min. Following this, 25 μL of butyrylthiocholine iodide (BTC, 15 mM) was added, and the absorbance was measured at 412 nm in 30-s intervals for 3 min. Tacrine, donepezil, and galantamine served as positive controls. All experiments were conducted in triplicate, the inhibitory activity against ChE (%) was calculated by the following Equation (10), and IC_50_ values were determined using GraphPad Prism 8.0 software

(10) $$\mathrm{Inhibition}\;\mathrm{rate}\;(\%)=(1-\frac{A}{B})\;\times \;100\%$$

where A and B refer to the enzyme reaction rate of sample and blank group, respectively.

Kinetic studies were conducted to measure reaction rates using the same methodology as in the enzyme inhibition experiments. In 96-well plates, varying concentrations of inhibitors (0, 10, 20, and 50 μM) were introduced, and the enzymatic reaction rates (v, △OD_417_ ·min^−1^) were recorded at different BChE concentrations (0.025, 0.05, 0.1, and 0.2 U·mL^−1^) with a fixed substrate concentration (1.5 mM). The relationship between reaction rates and enzyme concentrations was used to assess whether the inhibition was reversible. Additionally, reaction rates (v, △OD_417_ ·min^−1^) were measured at a constant BChE concentration (0.2 U·mL^−1^) while varying substrate concentrations (0.05, 0.1, 0.2, 0.4, 0.8 mM). A double-reciprocal Lineweaver–Burk plot of reaction rate versus substrate concentration was generated to elucidate the inhibition type based on the intersection points of the curves. Common inhibition types were identified as follows: competitive (intersects on the Y-axis), non-competitive (intersects on the X-axis), uncompetitive (parallel lines), and mixed (intersects in the second or third quadrant). Furthermore, by fitting the data to equations derived from the Lineweaver–Burk model, additional parameters such as the number of binding sites, the inhibition constant (K_i_), and the apparent coefficient (α) were determined based on Equations (11)–(13).

(11) $$\frac{1}{v}=\frac{K_{m}}{V_{\max}}\left(1+\frac{\left[I\right]}{K_{i}}\right)\frac{1}{\left[S\right]}+\frac{1}{V_{\max}}$$

(12) $$\mathrm{Slope}=\frac{K_{m}}{V_{\max}}+\frac{K_{m}\left[I\right]}{V_{\max}K_{i}}$$

(13) $$Y-\mathrm{intercept}=\frac{1}{V_{\max}^{\mathrm{app}}}=\frac{1}{V_{\max}}+\frac{\left[I\right]}{{{\alpha K}_{i}V}_{\max}}$$

where v is the reaction rate (∆OD_417_ꞏmin^−1^), [S] is the substrate concentration, [I] is the vasicine concentration, V_max_ is the maximum reaction rate (∆OD_417_·min^−1^), K_m_ and K_i_ represent the Michaelis–Menten constant and inhibition constant, respectively, and α is the apparent coefficient.

### 3.7. In Vitro Cytotoxicity and Neuroprotection Assay

Well-differentiated rat adrenal pheochromocytoma cells (PC12) and the corresponding culture medium (WHAA24F163) were obtained from Procell Life Technology Co., Ltd. (Wuhan, China). The culture medium was supplemented with 10% fetal bovine serum (FBS, 164210-50) and 1% antibiotics (a penicillin–streptomycin mixture). PC12 cells were seeded into sterile 96-well plates at a density of 7000 cells per well (100 μL) and incubated for 24 h in a humidified incubator at 37 °C with 5% CO_2_. Subsequently, the cells were treated with varying concentrations of the test compounds (10, 20, and 50 μM in 100 μL) and cultured for an additional 24 h. Cell viability was assessed using the 3-(4,5-dimethylthiazol-2-yl)-2,5-diphenyltetrazolium bromide (MTT) assay (Xavier, Wuhan, China), following protocols described in a prior study. Tacrine was employed as a positive control, and the cytotoxicity of the compounds was analyzed by comparing results from the treated groups with the blank and control groups.

To assess the neuroprotective potential of the compounds, an H_2_O_2_-induced injury model in PC12 cells was established. PC12 cells were seeded into 96-well plates and incubated for 24 h, as described previously. The cells were then pretreated with varying concentrations of the compounds (5, 10, 20, and 50 μM in 100 μL) for 1 h. Following pretreatment, the compound solutions were removed, and the cells were exposed to 100 μL of 400 μM H_2_O_2_ solution (3 wt.% in H_2_O, McLean, Shanghai, China) for 12 h. Cell viability was subsequently determined using the MTT assay. Tacrine was included as a positive control, while a model group, consisting of cells exposed to H_2_O_2_ without compound pretreatment, was used to evaluate the degree of cell damage caused by H_2_O_2_.

### 3.8. Spectroscopic Studies

***Fluorescence quenching experiment.*** The fluorescence intensity of the inhibitor–BChE complex was measured using a microplate reader with an all-black 96-well plate at three different temperatures (298, 304, and 310 K). The excitation wavelength was set to 280 nm, and the emission spectrum was scanned over the range of 320–460 nm with a slit width of 5 nm. The BChE concentration was maintained constant at 5 U·mL^−1^, while the compound concentration was varied in a gradient (0 to 50 μM). After mixing the compound and BChE, the samples were incubated at the designated temperature for 30 min prior to measurement. A PBS solution was used as a blank to account for background fluorescence and ensure accurate correction of the fluorescence intensity measurements.

***Time-resolved fluorescence spectroscopy experiment.*** The time-resolved fluorescence of free BChE (0.2 U·mL^−1^) and two groups of inhibitor–BChE complexes (with molecular concentrations of 40 μM and 80 μM, respectively) was measured at room temperature using a DeltaDiode system (280 nm, DD-280). The excitation wavelength was set to 280 nm (slit width 5 nm), while the emission wavelength was fixed at 335 nm (slit width 10 nm). Following the initiation of measurements, the fluorescence intensity decay over time was recorded. The resulting decay curves were analyzed and fitted using specialized software to determine the fluorescence lifetime. Typically, interactions between a compound and BChE can lead to changes in the fluorescence lifetime of BChE, providing insight into their binding dynamics.

***Three-dimensional fluorescence spectra experiment.*** The 3D fluorescence spectra of free BChE (0.2 U·mL^−1^), the inhibitor–BChE complex (inhibitor concentration of 50 μM), and the sample solution were recorded using a fluorescence spectrophotometer at 298 K. The excitation wavelength range was set from 200 to 400 nm, and the emission wavelength range was from 240 to 450 nm, with slit widths of 5 nm and 10 nm, respectively, and a wavelength increment of 5 nm. To eliminate interference from compound autofluorescence, the compound data were corrected by subtracting its blank signal. A comparison of the spectral characteristics, including changes in fluorescence peak position and intensity between free BChE and the complex, provides insight into the binding behavior of the inhibitor to BChE and its influence on the fluorophore’s microenvironment.

### 3.9. Molecular Dynamics Simulations

Molecular dynamics (MD) simulations were conducted for 50 ns using GROMACS 2019.5 [62]. The input file for the small molecule was prepared using AMBER force field atom types available on the ATB website (https://atb.uq.edu.au/index.py, accessed on 18 November 2024) [63]. The topology file for BChE was generated with the TIP3P water model and the AMBER14SB_parmbsc force field. The system was placed in a cubic simulation box containing 26,839 solvent molecules, ensuring a minimum distance of 1 Å between the protein and the box boundary. Sodium and chloride ions were added to neutralize the system, achieving a physiologically neutral state. Energy minimization of the complex was performed using the Polak–Ribiere conjugate gradient (PRCG) algorithm, with a time step of 1 fs and a convergence threshold set to a maximum force (Fmax) < 100 kJ/mol/nm.

Following energy minimization, the system was equilibrated in two phases: a 100 ps NVT equilibration and a 100 ps NPT equilibration, employing a V-rescale thermostat and a Berendsen barostat to maintain a temperature of 298.15 K and a pressure of 1 bar. During equilibration, constraints were applied to limit the length of hydrogen bonds. A final 50 ns MD simulation was then performed using a Parrinello–Rahman barostat with a time step of 2 fs.

The trajectories were analyzed using GROMACS (Gmx) scripts. Key parameters, including root mean square deviation (RMSD), root mean square fluctuation (RMSF), solvent-accessible surface area (SASA), and the number of hydrogen bonds, were evaluated to determine the stability and conformational transitions of the complexes throughout the simulation.

## 4. Conclusions

In this study, an integrated virtual screening strategy was employed to identify butyrylcholinesterase (BChE) inhibitors by combining machine learning-based QSAR modeling and molecular docking approaches. The XGBoost model exhibited high classification performance with an AUC of 0.9740, enhancing the efficiency of candidate selection. ADME property predictions and blood-brain barrier (BBB) permeability assessments further prioritized drug-like candidates. piboserod and Rotigotine were identified as potent BChE inhibitors, with IC_50_ values of 15.33 μM and 12.76 μM, respectively, demonstrating neuroprotective effects and low toxicity in PC-12 cells. Enzyme kinetic analyses revealed that piboserod acts as a mixed inhibitor, while Rotigotine functions as a non-competitive inhibitor. Fluorescence spectroscopy, molecular docking, and molecular dynamics simulations clarified their binding modes and interaction stability, with Rotigotine showing higher stability due to interactions with key residues such as His438 and Trp82. This study successfully identified two promising BChE inhibitors with anti-Alzheimer’s disease (AD) potential. Importantly, this is the first study to establish a link between Rotigotine and BChE inhibition, highlighting its strong safety profile and clinical translational potential as an ideal therapeutic candidate. Additionally, the activity assays and interaction studies provided a theoretical basis for further drug research and validated the feasibility of this screening strategy. This approach shows potential for drug screening and optimization targeting other diseases.

## Figures and Tables

**Table 1:** Average performance comparison of different models.

Model	Accuracy	AUC Score	Optimal Parameters
**RF**	95.36%	0.9728	‘max_depth’ = None; ‘min_samples_split’ = 5; ‘n_estimators’ = 200
**SVM**	94.64%	0.9677	‘C’ = 10; ‘gamma’ = ‘scale’; ‘kernel’ = ‘rbf’
**KNN**	94.09%	0.9721	‘n_neighbors’ = 7; ‘weights’ = ‘distance’
**XGBOOST**	94.94%	0.9740	‘max_depth’ = 9; ‘n_estimators’ = 100; ‘eval_metric’ = ‘logloss’

**Table 2:** The calculation results and information of the top 12 hits and positive control.

NO.	Name	Probability ^2^	Docking Score	Residue	Structure
1	Indacaterol ^1^	0.837774	−11.13	Trp82, His438, Glh197, Asp70, Phe329	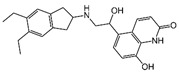
2	DC-05	0.631031	−10.846	Trp82, Asp70, Phe329, Trp231	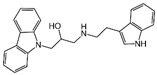
3	Z26395438	0.82116044	−9.39	Trp82, His438, Phe329	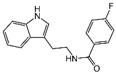
4	GSK-3β inhibitor 12	0.5691432	−8.696	Trp82, His438, Phe329	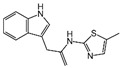
5	WAY-323062	0.89035976	−8.492	Trp82, Phe329	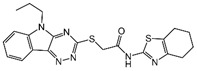
6	STM2120	0.69138443	−8.313	Trp82, Asp70	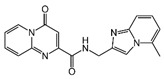
7	TM2-115	0.7532605	−8.107	Trp82, Phe329, Trp231	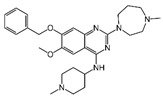
8	piboserod	0.7946475	−7.942	Asp70, Phe329, Trp231	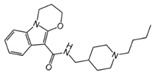
9	Metergoline	0.8255164	−7.937	Tyr332, Phe329	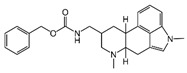
10	Rotigotine ^1^	0.75426644	−7.683	Trp82, His438, Tyr332, Trp231, Phe329	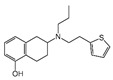
11	T16Ainh-A01	0.7628014	−7.548	Trp82, Phe329	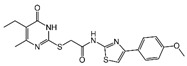
12	Iprindole	0.7948465	−7.54	Trp82, Tyr332	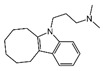
positive control	Tacrine	—	−8.040	Trp82, His438	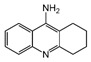
positive control	Donepezil	—	−7.914	Trp82, Tyr332	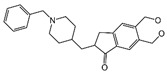
positive control	Galantamine	—	−7.142	Trp82	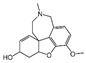

^1^ Marketed drug. ^2^ Predicted probability to be BChEls.

**Table 3:** In vitro BChE inhibitory activities of 12 hits.

NO.	Compounds	IC_50_ (μM) ^1^
1	indacaterol	n.a. ^2^
2	DC-05	n.a. ^2^
3	Z26395438	n.a. ^2^
4	GSK-3β inhibitor 12	n.a. ^2^
5	WAY-323062	n.a. ^2^
6	STM2120	n.a. ^2^
7	TM2-115	≈50
8	piboserod	15.33 ± 3.84
9	Metergoline	18.36 ± 3.29
10	Rotigotine	12.76 ± 4.22
11	T16Ainh-A01	n.a. ^2^
12	Iprindole	n.a. ^2^
positive control	Tacrine	0.14 ± 0.09
positive control	Donepezil	7.63 ± 1.21
positive control	Galantamine	15.62 ± 2.01

^1^ Data are the mean ± SD of three independent experiments. ^2^ not active at 50 μM (<50% inhibition).

**Table 4:** The quenching constant K_sv_, binding constant K_a_, binding sites n, and relative thermodynamic parameters for the interaction of piboserod and Rotigotine with BChE at different temperatures.

System	T (K)	K_sv_ × 10^3^ (L·mol^−1^)	R_a_^1^	K_a_ × 10^4^ (L·mol^−1^)	R_b_^2^	n	∆H (kJ·mol^−1^)	∆S (J·mol^−1^·K^−1^)	∆G (kJ·mol^−1^)
piboserod	298	5.250	0.998	8.595	0.999	1.052	−35.77	−44.61	−22.48
	304	4.720	0.999	6.724	0.999	1.035			−22.21
	310	4.250	0.995	4.912	0.999	1.014			−21.94
Rotigotine	298	2.64	0.999	5.084	0.998	1.066	−79.41	−195.34	−21.20
	304	2.25	0.998	2.896	0.997	1.022			−20.03
	310	1.54	0.999	1.469	0.999	0.975			−21.20

^1^ R_a_ is the correlation coefficient of the quenching constant Ksv. ^2^ R_b_ is the correlation coefficient of the binding constant Ka.

## Data Availability

Data are contained within the article/Supplementary Materials; further inquiries can be directed to the corresponding authors.

## Supplementary Materials

The following supporting information can be downloaded at: https://www.mdpi.com/article/10.3390/molecules30102093/s1, Figure S1: 2D interaction schematic of co-crystal ligand (A), Tacrine (B), Donepezil (C) and Galantamine (D) with BChE. Hydrogen bonds were shown as purple lines, salt bridge was shown as a pink violet gradient, π-π stacking interactions were in green lines, π-ion bonds were in red lines; Figure S2: Inhibition curves of piboserod (A), Metergoline (B) and Rotigotine (C); Table S1: ADME prediction results and BBB score of piboserod, Metergoline, Rotigotine and positive controls.
