# Supplementary material for: Discovery, Biological Evaluation and Binding Mode Investigation of Novel Butyrylcholinesterase Inhibitors Through Hybrid Virtual Screening
Source: Molecules. 2025 May 8;30(10):2093. doi: 10.3390/molecules30102093 (PMC12113681; doi:10.3390/molecules30102093)
Supplement: Supplementary file 1 [file molecules-30-02093-s001.zip › molecules-3583104-supplementary.pdf]

Supplementary Material

Di Discovery, biological evaluation and binding mode investigation of novel butyrylcholinesterase inhibitors through hybrid virtual screening

Lizi Li, Puchen Zhao, Can Yang, Qin Yin, Na Wang, Yan Liu* and Yanfang Li*

School of Chemical Engineering, Sichuan University, Chengdu, 610065, China

*Corresponding author, E-mail address: lyf471@vip.163.com (Y.Li)

*Corresponding author, E-mail address: yan_work@126.com (Y.Liu)

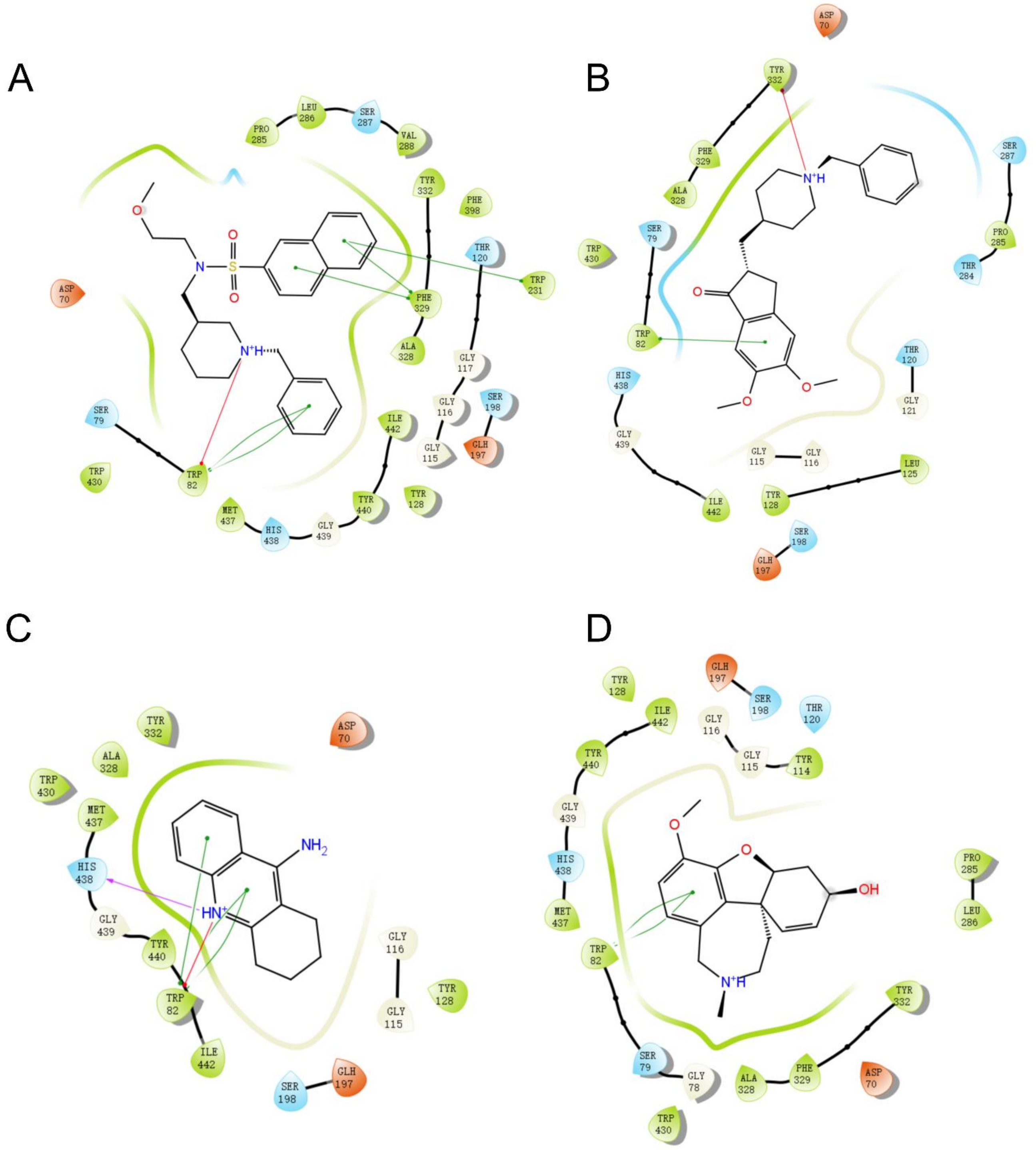

Fig. S1. 2D interaction schematic of co-crystal ligand (**A**), Tacrine (**B**), Donepezil (**C**) and Galantamine (**D**) with BChE. Hydrogen bonds were shown as purple lines, salt bridge was shown as a pink violet gradient, π-π stacking interactions were in green lines, π-ion bonds were in red lines.

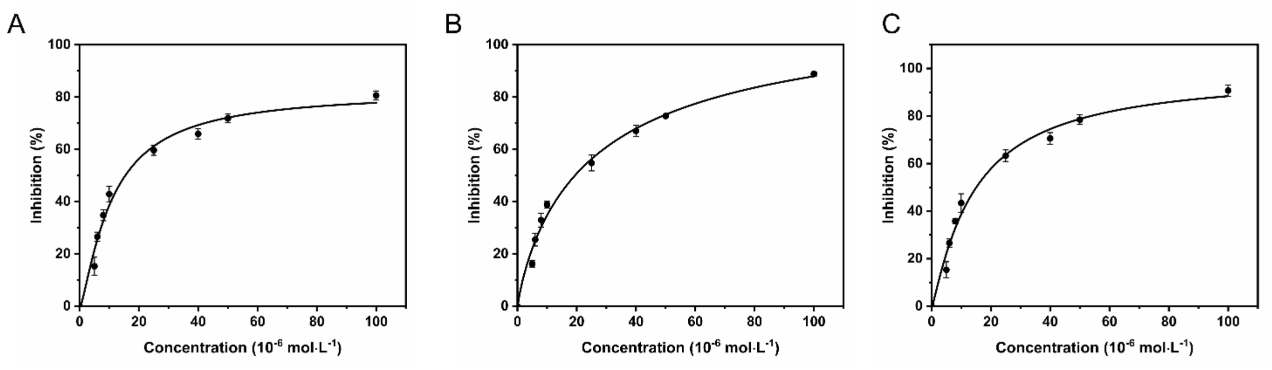

Fig. S2. Inhibition curves of Piboserod (**A**), Metergoline (**B**) and Rotigotine (**C**).

Table S1. ADME prediction results and BBB score of Piboserod, Metergoline, Rotigotine and positive controls.

**Predicted value**	**Piboserod**	**Metergoline**	**Rotigotine**	**Tacrine**	**Donepezil**	**Galanthamine**	**Recommended values**
CNS[a]	1	1	1	1	1	1	-2 (inactive) – 2 (active)
MW[b]	369.506	403.523	315.473	198.267	379.498	287.358	130–725
SASA[c]	695.202	692.752	617.477	429.227	631.268	506.928	300.0–1000.0
volume[d]	1262.402	1301.306	1086.067	707.891	1226.436	904.631	500.0–2000.0
donor HB[e]	1	1	1	1.5	0	1	0.0–6.0
accept HB[f]	5.5	4.5	2.75	2	5.5	5.2	2.0–20.0
QPlogPo/w[g]	4.329	4.942	4.583	2.579	4.055	2.037	-2.0–6.5
QPlogS[h]	-4.704	-5.366	-4.342	-3.109	-3.104	-2.179	-6.5–0.5
QPPCaco[i]	1134.09	575.912	880.201	2946.24	1057.394	794.128	<25 poor, >500 great
QPlogBB[j]	0.24	0.079	0.181	0.041	0.264	0.406	-3.0–1.2
metab[k]	1	5	6	3	6	4	1–8
QPlogKhsa[l]	0.715	1.119	0.746	0.066	0.452	0.028	-1.5–1.5
%HOA[m]	100	100	100	100	100	90.776	<25% poor, >80% high
PSA[n]	50.011	50.966	24.987	34.148	45.359	43.651	7.0–200.0
ROF[o]	0	0	0	0	0	0	Maximum=4
ROT[p]	0	0	0	0	0	0	Maximum=3
BBB Score[q]	4.23	4.09	5.62	5.38	4.53	4.57	4–6

[a] **CNS:** Predicted central nervous system activity. [b] **MW:** Molecular weight of the molecule. [c] **SASA:** Total Solvent Accessible Surface Area, in square angstroms, using a probe with a 1.4Å radius [d] **volume:** Total solvent-accessible volume, in cubic angstroms, using a probe with a 1.4Å radius. [e] **donor HB:** Estimated number of hydrogen bonds that would be donated by the solute. [f] **accept HB:** Estimated number of hydrogen bonds that would be accepted by the solute. [g] **QPlogPo/w:** Predicted octanol/water partition coefficient. [h] **QPlogS:** Predicted aqueous solubility. [i] **QPPCaco:** predicted apparent Caco-2 cell (a model for the gut-blood barrier) permeability in nm/sec.

[j] **QPlogBB:** Predicted brain/blood partition coefficient. [k] **metab:** Number of primary metabolites. [l] **QPlogKhsa:** Prediction of binding to human serum albumin. [m] **%HOA:** Predicted qualitative Human Oral Absorption. [n] **PSA:** Van der Waals surface area of polar nitrogen and oxygen atoms. [o] **ROF:** Number of violations of Lipinski's Rule of Five (molecular weight < 500, QPlogPo/w < 5, number of hydrogen bond donors < 5, number of hydrogen bond acceptors HB < 10). [p] **ROT:** Number of violations of Jorgensen's rule of three (QPlogS > -5.7, QPCaco > 22 nm/s, number of primary metabolites < 7). [q] **BBB Score:** Predicted blood-brain barrier permeability.
